# Conceptualising specialist supportive clinical management (SSCM): current evidence and future directions

**DOI:** 10.1186/s40337-022-00557-2

**Published:** 2022-03-07

**Authors:** Laura Kiely, Stephen Touyz, Janet Conti, Phillipa Hay

**Affiliations:** 1 Translational Health Research Institute, School of Medicine, Western Sydney University, Penrith, Australia; 2 University of Sydney InsideOut Institute, Camperdown, NSW Australia; 3 School of Psychology, Western Sydney University, Penrith, Australia; 4 Camden and Campbelltown Hospitals, SWSLHD, Campbelltown, NSW 2560 Australia

**Keywords:** Anorexia nervosa, Eating disorder, Evidence-based treatment, Person-centred therapy, Psychotherapy, Therapeutic alliance, Conceptualisation, Pedagogy, Meta-analysis, Theoretical integration

## Abstract

**Background:**

Current evidence-based treatments for adult anorexia nervosa (AN) have limitations, with high attrition, very poor outcomes for 20% of people, and no clearly superior manualised therapy for adults with AN. Specialist Supportive Clinical Management (SSCM) was designed as a control treatment but has evolved as a valid first line treatment. The present paper aims to provide an overview of the evidence base for SSCM and a pedagogical reconceptualization with expansion by theoretical integration (TI).

**Body:**

A secondary meta-analysis endorses SSCM as a promising treatment. This paper positions SSCM as a manualised therapy for adult AN with six unique features, namely (1) a philosophy which is person-centred, non-prescriptive, and informed by the person’s strengths and values, (2) a focus on the person through inclusion of *supportive psychotherapy* and problem (*clinical management*), within *target symptoms* as defined in relation to AN, (3) a flexible and responsive therapy that could be delivered by a variety of clinicians with experience treating AN (4) a commitment to reversing starvation though a directional approach and a defined yet flexible stance on dietetic intervention (5) a commitment to the therapeutic relationship within all three phases of treatment, and (6) a therapy ‘uncluttered’ by specific mandates. In addition, this paper positions SSCM as a treatment that may be strengthened by other modalities and may also be adapted to the treatment of other eating disorders (ED), not just AN. The level of therapist sophistication to deliver upon the supportive psychotherapy component is explored and future directions are offered.

**Conclusion:**

SSCM is a unique and valid first line treatment for AN and would benefit from further expansion in line with emerging understandings of AN to strengthen it as a treatment. Speculation on aspects of potency would benefit from further testing. The proposed re-conceptualisation of SSCM in the context of its evidence may strengthen it as a treatment overall, position it as adaptable for treatment of other eating disorders and make it more accessible to clinicians.

**Supplementary Information:**

The online version contains supplementary material available at 10.1186/s40337-022-00557-2.

## Plain English summary

The few established treatments for anorexia nervosa have limited success rates with high patient dropout. Specialist Supportive Clinical Management (SSCM) is a promising treatment for eating disorders. It would be beneficial to understand SSCM better and how and why it may work, which this paper proposes. SSCM is collaborative and flexible to people’s needs, offering them treatment choices and to progress at their own pace. It is less prescriptive than other manualised therapies and therefore could be offered to people more than once, building hopefulness. It requires an experienced psychotherapist to deliver it as SSCM addresses both the symptom of the ED (food and exercise behaviours) as well as life issues causing difficulty to a person (psychotherapy), as presented by the patient. Grounded in a sound scientific basis, SSCM is a promising treatment that would benefit from further development, in line with evolving understandings of AN, and some ideas for this are offered within the paper.

**Supplementary Information:**

The online version contains supplementary material available at 10.1186/s40337-022-00557-2.

## Background

The currently available evidence-based outpatient treatments for adult anorexia nervosa (AN) have consistently returned disappointing results whereby outcomes are poor and dropout rates are high [1–5]. In addition, there is a limited repertoire of endorsed treatments, all of which are manualized therapies and recent attempts to identify which of these may be a superior treatment for adult anorexia nervosa (AN) have failed e.g. Solmi et al. [6]. One of these treatments, specialist supportive clinical management (SSCM) differs in that it is not founded on a specific theoretical model and is without specialised intervention modules, yet it is found to be as efficacious as other therapies [6]. SSCM was first developed as an aetiological, atheoretical, manualised control psychological therapy for a three-armed treatment trial of underweight participants with AN [7]. In this seminal trial it was termed Non-Specific Clinical Management (NSCM). However, its relative efficacy particularly compared with interpersonal psychotherapy (IPT) but also cognitive behaviour therapy (CBT) lead to its renaming as SSCM.

SSCM differs from other manualised psychological therapies in not having a defined theoretical basis or prescriptiveness. It encompasses psycho-education (pro re nata), setting in collaboration with patients’ *target symptoms* for change (including weight regain), and *supportive psychotherapy* (SP). It is person-centred and therefore less directive than other therapies, demanding a sophisticated skillset of the clinician [8]. Although non-directive, SSCM is *directional* in the sense of moving toward change, in a patient-led way. It also requires therapists to have a specialist level expertise in treatment of anorexia nervosa. Session length and number are responsive to participant needs, where times range from 30 to 50 min. No “homework” is mandated by the therapist [9].

SSCM appears deceptively simple. A recent paper by Jordan et al. [10] sought to clarify misconceptions about SSCM [11–13]. However little attention was given to the sum of its many parts and how they interact to form the treatment “Gestalt”. SSCM demands clinician sophistication to deliver upon its SP orientation including an understanding of treatment nuance to clearly delineate from its exploratory psychotherapy cousins [14]. SSCM occurs on the polar end of a psychotherapy continuum, working with issues already conscious for the person. Attempts to bring previously unconscious material to awareness are not encouraged which is key to SSCM remaining pragmatic and directional. These processes may nevertheless arise and when material become conscious, this is addressed collaboratively and in ways that support the person’s autonomy and recovery.

Given expressed confusion about SSCM, a deeper understanding of its key therapeutic processes could inform the development of a conceptualised model. Such a model would enhance its understanding and potentially improve upon the integrity of its delivery for replication and testing. Furthermore, given the promise of this treatment and the time since development (1997) [15], it could be further expanded to reflect developments in the field [16] and contribute to the treatment repertoire for AN and possibly other EDs. The present paper thus aims to present a re-conceptualisation of SSCM in the context of its evidence, suggest a theoretical understanding for its efficacy and propose recommendations for further development, through theoretical integration.

## Main text

### SSCM: overview of the evidence

There is now substantive evidence that SSCM is a viable and efficacious psychological therapy for people with AN. There are four randomised controlled trials (RCT) comparing SSCM with an active psychological therapy [4, 7, 17, 18] as well as a trial of SSCM modified for treatment of severe and enduring anorexia nervosa (SEAN) (SSCM-SE;) [19]. SSCM-SE differs in having a focus on quality of life (QoL) improvement rather than mandatory weight regain. In 2015, we (PH, ST) published a review and meta-analyses of trials that had tested efficacy of psychological therapies as primary outpatient treatments for adults with AN [20]. In this review SSCM was treated as a control psychological therapy [7, 17, 18] and was not directly tested against other specialised and manualised psychological therapies in a single meta-analysis. A secondary analysis demonstrating the efficacy of SSCM as an active control compared to cognitive behavioural therapy (CBT), interpersonal therapy (IPT) and Maudsley anorexia nervosa treatment for adults (MANTRA) in reducing ED symptoms and improving weight, is presented in the attached online file (see Additional file 1). Furthermore, since this review, two network analyses [6, 21] have also supported the efficacy of SSCM as a first line psychological treatment in adults with AN whereby there were no significant differences found in terms primary outcomes—body mass index (BMI) [6, 21], clinical symptoms and dropout rate [6].

In addition to these studies in AN [4, 7, 17–19] Robinson and colleagues [22] have conducted a transdiagnostic RCT with SSCM as a comparative therapy in a study of 68 people with an ED (6% with AN) and personality disorder. Despite SSCM having 70 percent fewer sessions (Mentalization Based Therapy 88 sessions and SSCM maximum 26 sessions) and limitations of the trial including high follow up attrition (40%), there were no between group differences found in the main outcomes.

### Towards a better understanding of SSCM

SSCM thus has a robust evidence base and provides an alternative grounded in *supportive psychotherapy* principles as described by Dewald [14]. An essential element of SSCM is to take time and position the person as central through a patient-led, strengths based and values-driven orientation in which the patient sets the pace for change. The lack of treatment clutter allows room to explore problems before prematurely fixing them and positions the patient as change agent [10]. The elements of pace, space and empowerment through a person-centred orientation should not be overlooked. This leans into established psychotherapy wisdom regarding change [23–25] and is consistent with what patients describe as helpful to recovery [26] and is aligned with contemporary patient-centred, recovery-orientated mental health care [27]. This may account for SSCM’s potency. Person centred therapy (PCT), originally proposed by Rogers [28] represented a departure from psychotherapy conventions in which the therapist was seen as the expert. Instead, PCT is a patient-led, empathic approach that holds the belief that a person has inherent capacity for growth and motivates and empowers the person to move in the direction of change. Furthermore, a key distinction of SP is that it considers issues that are presently in the person’s conscious awareness, thereby maintaining treatment direction and focus.

**Fig. 1:**
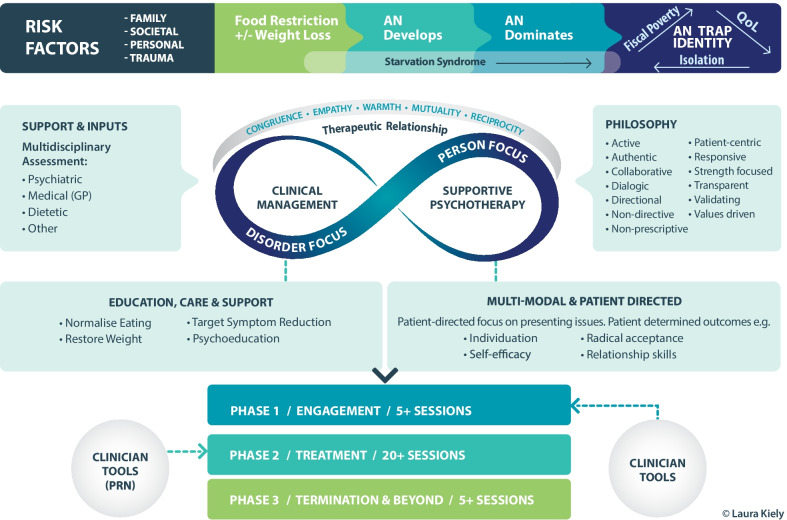
Reconceptualisation of SSCM

Alongside the SP sits *clinical management (CM)* of *target symptoms* the model defines as related to AN, a notion adapted from Joyce 1995 [29]. This renders the treatment a directional one with a treatment non-negotiable of normalizing eating to reverse starvation as a known maintaining factor of AN (see Fig. 1). The delicate tension of establishing the treatment non-negotiable is supported within skilful person-centred care of SP with particular attention in phase one *orientating to treatment* (See Fig. 1). The concurrent and distinct focus on the person and the problem not only serves to externalise AN but also potentially strengthens a person to build their life without AN, by addressing life issues as they present by the individual. It is reported that patients with EDs find it helpful to be ‘seen and treated as a whole person’ [26] p. 586 and that it is experienced as unhelpful to focus exclusively on weight and eating symptoms [30]. While patients consider structure to treatment as supportive, care needed to be individualised, flexible, engaging and personal to be accepted by patients [31]. The compatibility of SSCM with patient preferences, through its structured yet flexible and individualised nature together with concurrent symptom and person focus may account for its efficacy.

### The therapeutic alliance

A further strength to this wrap-around treatment is support within a therapeutic relationship or alliance, whereby the therapeutic alliance (TA) is a non-specific therapeutic factor. The therapeutic alliance is broadly defined by Norcross and Lambert [32] p304 as ‘*the feelings, and attitudes that the therapist and client have towards one another, and the manner in which these are expressed*’. The TA is a complex concept and measured in varied ways [32, 33]. Its effects may vary for diagnostic groups, age and therapy types but are significant.

There has been limited attention to the TA as it relates to ED treatment. A meta-analysis of 20 studies by Graves et al. [33], using a range of ED diagnoses and treatment modalities used temporal analysis to measure effect size of the quality of the therapeutic alliance (TA) on outcomes (weight and ED cognitions) at different time points. They found that early symptom improvement was related to early to mid TA measures with low to moderate effect size, however causality is not implicated, but rather a bidirectional relationship between the two variables. Significant moderators included age, type of ED diagnosis and treatment type, where the TA was more important in predicting outcomes for younger patients, those with AN compared with other EDs and possibly in treatments which view the therapeutic relationship as an aspect of potency with the example of greater importance in supportive psychotherapy compared with CBT. There are several significant limitations, including small number of studies with small sample sizes and the use of 9 different measurement tools for TA, instrumented by different groups e.g. patient, therapist or parents. Thus, there is more to learn about the contribution of TA to outcomes and with regards to different therapy modalities [6].

The qualitative ED literature consistently reports that patients value the therapeutic relationship in treatment as supportive to recovery [26, 30, 31, 34]. In the most recent of these meta syntheses Karlson et al. [31] offers a patient-constructed definition of therapist qualities and relationship attributes that patients experience as helpful. Therapist qualities included empathy, flexibility, patience, acceptance, authenticity, accurate listening, emotional accessibility, and transparent and clear communication. Patients needed time to build a trustful and secure care relationship (> 6 months) and it was important they felt that the therapist will remain regardless of the treatment’s success by way of instilling hopefulness.

The spirit of SSCM is dedicated to the therapeutic relationship throughout the processes of all three phases of its treatment, viewing it as intrinsic to all aspects.

### Therapist sophistication and the therapeutic relationship

While the model appears straight forward, SSCM requires the clinician delivering the treatment to be a skilled counsellor to ensure the person is positioned as central in the therapeutic process [28]. To effectively deliver this model within a relational framework, the therapist is required to be an active person in the treatment who skilfully uses role modelling and self-disclosure. For example, in response to a patient who offers an interpersonal dilemma within their SP, the therapist may appropriately use self-disclosure in describing an approach to interpersonal difficulties by setting boundaries. This would be congruently modelled within the therapy relationship, by way of role modelling. SSCM has a specific view about transference and negative transference in the therapeutic relationship. The therapist is required to be astute to negative transference early on and this is not explored, which serves to maintain treatment direction. Rather, transference is named, addressed in a way that will not challenge a person’s defences (build resistance), to support the therapeutic relationship, demonstrate that the therapist is comfortable and accepting of the patient’s negative feelings and to promote change [15].

The therapist assumes the person to be the expert on themselves and encourages them to mobilize their own capacity for change, in line with what is important to them. The therapist’s focus is on evocation by reflecting and exploring and this is required to take time. The commitment to the therapeutic relationship may account for some of its potency [35] and good treatment retention (76%) as found in the Touyz et al. SSCM-SE trial [19]. Furthermore, patient treatment preferences have affirmed its importance [36].

In the delivery of SSCM, the therapist themselves also need to have capacity to tolerate its relatively less structured and uncertain nature [37] and discharge themselves from the role of ‘expert’, be comfortable within a directional but flexible therapy stance [38] and bring creativity in what could be framed as the “art” of psychotherapy. As a distinction to other models the therapist needs to be able to put aside their own agenda, be prepared to work slowly, be self-reflective, flexible and responsive in their delivery of therapy and fully invested in the patient’s individuation and their own planned redundancy. This nuanced work is that of an experienced clinician. Its PCT orientation may also render the treatment suited to delivery by counselling orientated mental health practitioners, representing its adaptability to a range of clinicians.

Given the authors of SSCM’s speculation about the therapeutic relationship as an aspect its potency [10] and its contribution to outcomes particularly with AN and individual psychotherapies such as SSCM [33] as well as being important to patients, an overt description of the key qualities to support this are offered within the proposed conceptual model in Fig. 1.

### Person centred dietetic intervention

In SSCM dietetic support is offered in a flexible way that is responsive to individual needs—be it to inform part of the assessment phase or included alongside the treatment intervention or the dietitian as consultant to the primary therapist. It holds a person’s past ability to feed themselves or others to example by way of building upon existing capacity. The dietetic principles and orientation for normalizing eating within SSCM are uniquely defined. It offers a ‘broad brush strokes’ approach with its focus on dietary Variety and Regularity and as well as Adequacy (which could be better described conceptually as the “VRA model to dietetic intervention”) with supporting clinician resources as needed (see Fig. 1). A closer focus on the adequacy of intake may be required where *target symptoms* are not adequately progressing to wellness. As an extension to this VRA model, there are more comprehensive iterations of this type such as the Regularity, Adequacy, Variety, Eating Socially and Spontaneity (RAVES) model [39], which is an obvious inclusion within an updated iteration of SSCM.

A study of content analysis has shown the focus on *clinical management* within SSCM to account for 80% of the therapy content [40]. This was seen as a negative by the therapists delivering it. Given the weighting towards clinical focus on education, care and support to provide psychoeducation by way of normalising eating to reduce target symptoms (see Fig. 1), it can be inferred that a specialist ED dietitian is an essential member of the treatment team. A dietitian has background training in medical management as it applies to this treatment and can accurately relate changes in eating back to *target symptoms* [41]. In contemporary dietetic practice and especially when working in ED treatment, patient-centred care (PCC) and counselling skills (including motivational interviewing) are endorsed as part of an Accredited Practising Dietitians (APD) skill-set [41]. Therefore, the applicability of SSCM for APDs with requisite ED experience in a contemporary understanding of dietetic training and practice, warrants further investigation.

In the context of its flexible and person-centred orientation, there is also the possibility to engage the minutiae of food and nutrition with meal plans and self-monitoring to best serve the individual, per the patient therapist collaboration [42]. These aspects frame SSCM as a specific, strength-focused, values orientated, patient driven and responsive treatment.

Further consideration about the practicalities of translating this model from the clinical trial to treatment setting are required in order to adapt SSCM to the treatment setting and are shown in Fig. 1 as *supports and inputs* as well as *clinician tools.*

### SSCM and theoretical integration

A more clearly defined theoretical framework as proposed by Fig. 1, may enhance the clinical utility of the intervention. Thus, SSCM could be further strengthened as a treatment by expansion through theoretical integration (TI). The past few decades have seen a shift towards TI more broadly in psychotherapy [43, 44] whereby TI is defined by Castonguay et al. 2015 p. 366 [45] as; *‘the integration of theories and techniques of two or more psychotherapies into a new conceptualisation of change or treatment approach’*. This is distinct from ‘eclecticism’ [46] which borrows ‘techniques’ without attention to how they fit within a revised conceptual model and underpinning theories.

A limitation to the expansion of SSCM in this context is that it was developed as an atheoretical control therapy [10]. Thus, it does not currently have a defined theoretical basis, being a pre-requisite for TI and integrative efforts could be misconstrued as eclectic. Aspects of SSCM’s potency may be the synergy of multiple factors. These may include; the implicit person-centred orientation (supported by lack of treatment mandates such as homework and specific modules allowing flexibility, time and responsiveness and also the collaborative formulation of target symptoms), a pragmatic and directional (as distinct from directive) orientation whereby the therapy maintains direction in a patient-led way. For example, when consistent with a patient’s goals, the therapist may ask ‘what are your best reasons for improving some of your food choices, in a way that you feel able?’ SSCM also has a commitment to the reversal of starvation as a known maintaining factor, comprehensive bio-psychosocial assessment including comorbidity (which may require additional treatment), the therapeutic alliance in which specific attention is given to minimize patient defences or resistance, multidisciplinary inputs, psychoeducation (pro re nata) and the concurrent symptom and person focus. Within the proposed expansion of SSCM in Fig. 1, other attributes could add further strength to its theoretical basis. For example, attention to the aetiological trajectory over the course of AN, inclusion of motivational strategies through theoretical integration with MI, incorporation of risk factors to guide treatment focus, and further considerations to enable translation to the multidisciplinary treatment setting. Factoring all of the above and combined with SSCM’s relative efficacy, a theoretical basis can be conceptualised.

### Proposed theoretical basis for SSCM

SSCM adopts a person and a symptom focus. It is a directional therapy, whereby eating disturbances are considered coping strategies, enlisted by a person to help them with life difficulties. The purpose of psychoeducation and motivational strategies are to support a person to make an informed decision about their ongoing commitment to engaging in ED behaviours. That is, to recognize for themselves, that the eating disturbance, while immediately comforting may lead them to make choices that are not compatible with their life values and which if they remain committed to, may lead to an impoverished life. This is because eating disordered behaviours are a self-perpetuating maintaining factor (complicated by their endorsement by society), and this is the rationale for the expanded aetiological model—the AN or ED “trap” (see Fig. 1). SSCM is delivered in a form that is cognizant of perfectionistic traits it names as a risk factor for AN. While it does not directly address these traits—it could be argued that setting targets and goal setting may encourage perfectionism. Therefore, progress is emphasised to be slow and steady rather than rapid and impressive, to promote ongoing self-efficacy. The therapist offers their full support to remain alongside and committed to the person in an empathic, non-judgmental, and encouraging way, via a therapeutic alliance, as the person navigates their way out of an eating disorder, which may take time. The therapist holds belief in the person’s capacity for healthy functioning (patient-centred and recovery orientated care [27, 47]) and is cognizant that the ED is likely to be highly valued by the patient and careful attention is paid to minimizing defenses. Additional psychological therapy drawing from other theoretical modalities e.g. psychodynamic, cognitive-behavioural may be required to support long term recovery from an eating disorder, which is known to have varied and complex causes. That, which may not be in the immediate, conscious awareness for the person—be it trauma (relational, emotional, physical or sexual abuse) to the spectrum of family, societal and personal risk factors (see Fig. 1). On this premise, therapy may continue beyond the 20–40 focused sessions. A person is encouraged to take a compassionate and non-dichotomous view of themselves and their treatment progress [48] where relapse is seen as an opportunity for the development of further insight and skills in overcoming the ED and a signal that ongoing support in the recovery from an ED is usually needed, so as to retain hopefulness always.

### Theoretical expansion of SSCM to incorporate strengths of other modalities

SSCM, within the proposed conceptualisation can be understood as a humanistic and relational therapy, rendering it consistent with aspects of motivational interviewing [49] and PCT [28] and could be enhanced by incorporating them more comprehensively. For example, the spirit of motivational interviewing (MI), including partnership and collaboration, respect of personal autonomy and acceptance of the person (not the problem) aligns with that of SSCM. Given the philosophical harmony between SSCM and MI, it could be further adapted and expanded to include other potent aspects of MI regarding motivation and ambivalence. This would also address the authors expressed limitation of the therapy as lacking in motivational techniques [10]. While it does ‘*use encouragement, awareness of physical and psychosocial costs of AN and of desired areas of life change’* (Jordan 2020 p 158) this could be further enhanced by incorporating MI more comprehensively given philosophical harmony. Psychotherapy integration has the potential to contribute new treatment directions, which are much needed in eating disorder treatment, without replicating the work of other established therapies [43].

Founded on solid research and theoretical basis [25] MI offers a diverse and sophisticated skillset with emphasis on the language to support evocation and minimise defences such that a person can generate their own ideas for a better life [50]. Change is elicited from within the person, on their terms, where they learn what they believe, including what they give value to, when they hear themselves speak [49]. In a systematic review on the use of MI in the treatment of EDs [51], the authors concluded (pp. 10) that *‘there is potential for using MI in the field of eating disorders, particularly with respect to readiness for change*. Thus, evidence points to MI as a promising inclusion in ED treatment, a condition which is considered ego-syntonic and therefore met with ambivalence and resistance to change.

Keeping with the spirit of the flexible and responsive attribute of SSCM is significant considering the therapist is standing with the person in getting their life back from a disorder with control at its core [52]. In the Mosaic trial [17], the SSCM group improved more in terms of reduced obsessionally on the Obsessive Compulsive Inventory, than in the MANTRA arm—so SSCM may be a therapy that enables more flexibility by being flexible of itself.

Insisting on rigid adherence to a single therapeutic orientation that ignores the possibility of expansion to include potent elements of other therapies is a treatment disservice and may limit the full potential of SSCM. In the original trial for SSCM [7], therapy content was dictated by patients, however for research integrity, therapists were constrained to avoid specific strategies or foci of IPT or CBT [15]. In a practice setting, enlisting efficacious elements of CBT and IPT to best serve the person is in keeping with the progressive and contemporary notion of TI. This is at the very essence of PCT [28]. For example, if a person identifies eating episodes in response to adverse emotions, the use of monitoring to increase awareness about habitual eating may be of benefit to that individual. This would fit within the person-centred and responsive conceptulisation of SSCM and also recognises the probable importance of specific treatment factors for specific disorders.

Furthermore, looking to one treatment as providing all the answers to a complex illness on a continuum [53, 54] where we do not have studies endorsing treatment specificity (which treatment for which person and when), may do patients a disservice. Instead, with a more clearly defined conceptual framework as proposed in Fig. 1 and a defined theoretical basis, SSCM has scope to remain true to its philosophies and flexibly tailored to the needs and preferences of a person living with AN and the therapist delivering it, within a treatment non-negotiable framework that also prioritises their safety. This is in keeping with others who have highlighted the importance of listening to a person’s psychotherapy treatment preferences out of respect for their ‘rights and dignity’ and to optimise treatment outcomes [23].

### Possible strengths and potency of SSCM—the ‘Tortoise and The Hare’

SSCM has shown promise as an adaptable treatment across the spectrum of eating disorders to long-standing illness (SEAN) [55] where the outputs for the CM component have been successfully updated to include a QoL focus [19]. SSCM has also shown transferability to other EDs not just AN [22]. Hence, the conceptual model for SSCM can be used to frame the therapy, which may also be adaptable to other EDs by modifying the agreed target symptoms, within CM outputs.

In the absence of a clinician led agenda for homework and monitoring expectations, SSCM by its nature is easier to adapt the pace to be person-centred and responsive. The slower pace also addresses the parallel process of anxiety where clinicians may take up the person’s anxiety [49] to coerce change. In this context, clinicians may also seek to fix the illness, which is frequently resisted by the person with a lived experience and poses a risk to the therapeutic relationship. SSCM gives the clinician and the person explicit permission to take their time. Limited qualitative research of treatment experiences literature suggests that coercive, hurried and “done to” attributes as negatively impacting on treatment adherence, drop-out and relapse [36]. The person-centred underpinning within SSCM may produce a slower but more sustained change and further studies could confirm this.

In recognition of the potential benefit of taking time as shown in Fig. 1 allocation to phases one to three may be denoted 5 +, 30 + and 5 + sessions, respectively. These time suggestions are to make it practical in the context of a forty-session treatment model, but should again be responsive, considered as a minimum timeframe and further research into ideal length of treatment could better inform this.

### Translating SSCM into clinical practice and future directions

SSCM has not been adequately translated to the clinical treatment setting but does lend to a multidisciplinary treatment team whereby the primary therapist is the co-ordinator of the complex medical care needs in collaboration with the person’s doctor. An attempt to incorporate these contributions to the multi-care needs of individuals with AN in a treatment setting has been added to Fig. 1 under the heading ‘*Supports and Inputs’.*

In this conceptulisation, the philosophy of SSCM incorporates foundational influences of SP and CM [14, 29]. In SSCM patients experience a therapeutic climate which encourages their individuation as they are empowered to make decisions about their own treatment and recovery path. Modelling this with a treatment that knows clearly what it is, is paramount in remaining true to its theoretical origins [14]. As such, some of the key philosophies and spirt of SSCM (e.g. active, authentic and collaborative) are suggested in Fig. 1.

To enhance adaptability to individual clinicians, the SP component of this model could be re-defined more broadly as multimodal, patient-directed therapies—as per the expanded Fig. 1—for example Person Centred Therapy [28]. The SP aspect of this treatment, which is informed by the early (1994) work of Dewald [14] could be further refined and defining features are offered in Fig. 1 ‘philosophy’. The proposed iteration in this paper (see Fig. 1) includes subheading ‘multi-modal & patient-directed’ with a description and outputs described as person-directed focus on presenting issues—patient determined outcomes, for example: individuation; self-efficacy; radical acceptance; and relationship skills, to be more practical for clinicians.

We propose an expanded aetiological model of SSCM, adapted from Hay et al. 2016, to include a more cyclical end-point [56] Given further understanding about AN and identity formation within the qualitative literature [57–59], an extension to Hay’s conceptual modal is proposed in Fig. 1 to include *‘AN Identity Trap’*. This Figure could also be further expanded to explicitly acknowledge trauma as an aetiological maintaining factor [60] requiring specific evidenced based treatment as identified at the pre-treatment assessment phase see ‘Risk Factors’ in Fig. 1.

The adaptability of SSCM for the treatment of other eating disorders is plausible, given that within a transdiagnostic understanding all EDs share commonalities in symptoms (except for weight loss and emaciation). SSCM sets target symptoms in relation to and in collaboration with the individual patient and has shown adaptability to include other outputs such as quality of life [19]. The underpinning aetiological conceptualisation of SSCM would require further consideration to support this, however based on theoretical suitability, testing in other ED populations is indicated.

The place of relapse and recovery within treatment could be accepted as a clinical reality for a condition in which recovery may take time [1, 6] as seen in the phase 3 of treatment of Fig. 1, whereby ‘beyond’ is added to the ‘termination’ phase. Acknowledging that relapse may be part of ongoing recovery may prevent perceived treatment failure and instil hope.

As the content of SSCM is not prescribed, new therapeutic directions may emerge by reemploying it as a modality or by promoting ongoing engagement in exploratory psychotherapies once target symptoms are reversed. SSCM may not be an end point.

Hence, SSCM is positioned as part of an expanded treatment picture, proposed to best serve the individual and their unique presentation of AN. In summary, we propose an expansion of the aetiological SSCM AN model to include the “AN Trap” (see Fig. 1) as an endpoint underpinned by starvation syndrome; inclusion of trauma as a risk factor to development of AN and addressing this in the assessment phase; refinement of the various aspects of the treatment to be more clearly defined and conceptualised and finally, acknowledging that SSCM treatment is not an end point for all. SSCM may be reemployed and or more exploratory psychotherapies enlisted beyond SSCM treatment and accepted as part of the treatment course to retain hopefulness. Exploration of the efficacy for other eating disorders is also warranted.

## Conclusion

The present paper has proposed a first step in a re-conceptualisation of SSCM as an evidence-based treatment within a revised model as presented in Fig. 1. Whilst the potency of SSCM therapy is incompletely understood, as outlined in this paper there are putative factors at play, such as SSCM’s unique approach to dietetic intervention, treatment goals, investment in the therapeutic relationship within all phases of treatment and it’s directional but patient-driven orientation. Further consideration could be given to the therapeutic relationship; and re-framing SSCM with theoretical integration to incorporate complimentary therapeutic aspects of therapies such as MI and PCT, by way of supporting patient-driven outcomes.

## Supplementary Information

**Additional file 1**. Secondary Analysis.

## Data Availability

Not applicable.

## Abbreviations

AN: Anorexia Nervosa
CBT: Cognitive behaviour therapy
CBT-E: Cognitive behaviour therapy-enhanced
CM: Clinical management
IPT: Interpersonal psychotherapy
MANTRA: Maudsley Therapy for Adults with Anorexia Nervosa
MI: Motivational interviewing
PCT: Person centred therapy
PCC: Patient centred care
RAVES: Regularity, Adequacy, Variety, Eating Socially and Spontaneity
RR: Risk ratio
S/MD: Standardised /mean difference
SE: Severe and enduring
SEAN: Severe and enduring anorexia nervosa
SP: Supportive psychological therapy
SSCM: Specialist supportive clinical management
TI: Theoretical integration
VRA: Variety and regularity and adequacy
WHO: World health organisation
