# Supplementary material for: Conceptualising specialist supportive clinical management (SSCM): current evidence and future directions
Source: J Eat Disord. 2022 Mar 7;10:32. doi: 10.1186/s40337-022-00557-2 (PMC8900319; doi:10.1186/s40337-022-00557-2)

**Additional file: Secondary meta-analysis of Specialist Supportive Clinical Management (SSCM) in randomised controlled trials of outpatient psychological therapies in adults with anorexia nerosva.**

In this piece, we report findings from a secondary meta-analysis of a systematic review (1) where SSCM is compared to other manualised specialist therapies. Three outcomes were chosen based on clinical and diagnostic relevance, namely weight (or BMI kg/m^2^) and eating disorder symptom severity as measured by the Eating Disorder Examination interview (2) and an indicator of therapy acceptability, namely treatment attrition. A systematic search of the Cochrane Databases of trials and other databases MEDLINE (1950-), EMBASE (1974-) and PsycINFO (1967-); quarterly searches of the Cochrane Central Register of Controlled Trials (CENTRAL); LILACS, and International Trial Registers (ClinicalTrials.gov and the WHO trials portal (ICTRP) for all randomised controlled trials of any psychological therapy compared to SSCM in the outpatient treatment of adults (persons aged 16 or more years) with AN was conducted to July 2014 in the preceding published review, and for the purposes of this paper updated to June 2018. A quality appraisal of trials utilised the Cochrane risk of bias tool (3). The risk ratio (RR) using a random-effects model was employed for binary outcome data as a more conservative statistic than the odds ratio, and appropriate where the outcome is not a rare event such as death. Where studies used the same outcome measured on the same scale, we used the mean difference (MD) and used standardised mean difference (SMD) analyses for continuous outcome data where there was variability in outcome measures. All results are reported with 95% confidence intervals (CIs) for RRs. A random-effects model was employed a more conservative statistical measure (section 9.3.4.3, Higgins (3)) to mitigate a Type I error where there were small numbers of trials. For the purpose of this analysis, where there were studies with multiple intervention arms and one SSCM arm we included each pair-wise comparison separately, but with shared intervention groups divided out approximately evenly among the comparisons. For dichotomous outcomes, both the number of events and the total number of patients were divided up. For continuous outcomes, only the total number of participants were divided up, and the means and standard deviations left unchanged.

The results of this secondary analysis were as follows: Five published trials had data available for inclusion of trials of SSCM compared to another specialist psychological therapy. The first trial compared Interpersonal Therapy (IPT) and Cognitive Behaviour Therapy (CBT) to SSCM (4). Two further trials have compared the Maudsley Therapy for Adults with Anorexia Nervosa (MANTRA) to SSCM (5, 6) one compared SSCM with CBT – Enhanced (CBT-E) and MANTRA (7), and one compared CBT-Severe and Enduring (SE) to SSCM-SE (8). The trials used variable intensity and number of sessions, from 20 weekly sessions (4) to between 20 and 30 weekly sessions with four once monthly follow-up sessions and 34 weekly sessions over 8 months (8). All trials had an adequate method of randomisation and allocation concealment. All had risk of bias from lack of blinding of participants to therapy group, but all had blinding of outcome assessment and in other respects the trials were rigorous. They all used an intention-to-treat approach and gave information on participants who did not complete therapy, assessed therapist fidelity to treatments and reported short, and longer-term follow-up of participants.

As shown on Table 1 and the Forrest Plots there were no differences that reached significance between SSCM and another therapy for weight gain (Body Mass Index) or for reduced symptom severity. In the pooled meta-analysis MANTRA was favoured over SSCM with regards to treatment attrition. There were insufficient trials of IPT versus SSCM for pooling of data, but IPT was found to have less reduction in EDE Restraint scores compared to SSCM. The meta-analyses were limited by the small numbers of trials, only one trial of SSCM compared to IPT, and the division of pairwise comparisons from the McIntosh et al. (4) and Byrne et al. (7) trials (this method only partially overcoming known unit-of-analysis error because the resulting comparisons remain correlated (Higgins, section 16.5.4, 3). One trial (8) also was of severe enduring anorexia nervosa where the primary outcomes were not those investigated in this paper. Whilst SSCM may be less acceptable than MANTRA, it can be concluded that SSCM is an appropriate therapy to use, particularly where CBT or MANTRA are not accessible or where access may be delayed.

**References:**

1. Hay PJ, Claudino AM, Touyz S, Abd Elbaky G, Hay PJ. Individual psychological therapy in the outpatient treatment of adults with anorexia nervosa. Cochrane Db Syst Rev. 2015;2018(2):CD003909-CD.
2. Fairburn CG, Cooper Z, O’Connor M. Eating Disorder Examination (Edition 16D) in Fairburn CG. Cognitive behavior therapy and eating disorders., New York: Guilford Press; 2008; pp 265-308..
3. Higgins JPT, Green S (editors). Cochrane Handbook for Systematic Reviews of Interventions. Chichester: John Wiley & Sons, 2011
4. McIntosh VVW, Jordan J, Carter FA, Luty SE, McKenzie JM, Bulik CM, et al. Three Psychotherapies for Anorexia Nervosa: A Randomized, Controlled Trial. Am J Psychiatry. 2005;162(4):741-7.
5. Schmidt U, Oldershaw A, Jichi F, Sternheim L, Startup H, McIntosh V, et al. Out-patient psychological therapies for adults with anorexianervosa: randomised controlled trial. Br J Psychiatry. 2012;201(5):392-9.
6. Schmidt U, Magill N, Renwick B, Keyes A, Kenyon M, Dejong H, et al. The Maudsley Outpatient Study of Treatments for Anorexia Nervosa and Related Conditions (MOSAIC): Comparison of the Maudsley Model of Anorexia Nervosa Treatment for Adults (MANTRA) With Specialist Supportive Clinical Management (SSCM) in Outpatients With Broadly Defined Anorexia Nervosa: A Randomized Controlled Trial. J Consult Clin Psych. 2015;83(4):796-807.
7. Byrne S, Wade T, Hay P, Touyz S, Fairburn CG, Treasure J, et al. A randomised controlled trial of three psychological treatments for anorexia nervosa. Psychol Med. 2017;47(16):2823-33
8. Touyz S, Le Grange D, Lacey H, Hay P, Smith R, Maguire S, et al. Treating severe and enduring anorexia nervosa: a randomized controlled trial. Psychol Med. 2013;43(12):2501-11.

**Table: Results of meta-analyses comparing Specialist Supportive Clinical Management (SSCM) with two other psychological therapies.**

Comparison Studies Pooled Analysis (95% CI)

SSCM vs all other

BMI 5 trials, 451 participants NS, SMD -0.05 (-0.24, 0.14)

EDE score 5 trials, 451 participants NS, SMD -0.18 (-0.57, 0.21)

Treatment attrition 5 trials, 452 participants NS, RR 1.23 (0.97, 1.56)

SSCM vs MANTRA

BMI 3 trials, 273 participants NS SMD -0.05 (-0.29, 0.19)

EDE global score 3 trials, 273 participants NS SMD 0.12 (-0.28, 0.51)

Treatment attrition 3 trials, 274 participants 52% vs 40% RR 1.33 (1.02, 1.74)

SSCM vs CBT

BMI 3 trials, 149 participants NS SMD -0.12 (-0.45, 0.22)

EDE global score 3 trials, 149 participants NS SMD -0.23 (-0.66, 0.20)

Treatment attrition 3 trials, 149 participants NS RR 1.03 (0.60, 1.75)

Notes:

There were insufficient trials for data pooling for comparison of SSCM vs IPT.

In the trial of CBT or IPT versus SSCM (4) only sub-scale EDE scores were available.

In the trial of SSCM vs CBT-E and MANTRA (7) only mean differences in BMI were available

BMI = Body mass index (kg/m^2^), NS=not significant, RR=risk ratio, S/MD=standardised /mean difference, EDE=Eating Disorder Examination, MANTRA = Maudsley Therapy for Adults with Anorexia Nervosa , CBT = Cognitive Behaviour Therapy

**Forest Plots**

1. **SSCM versus another therapy, Body Mass Index outcome.**


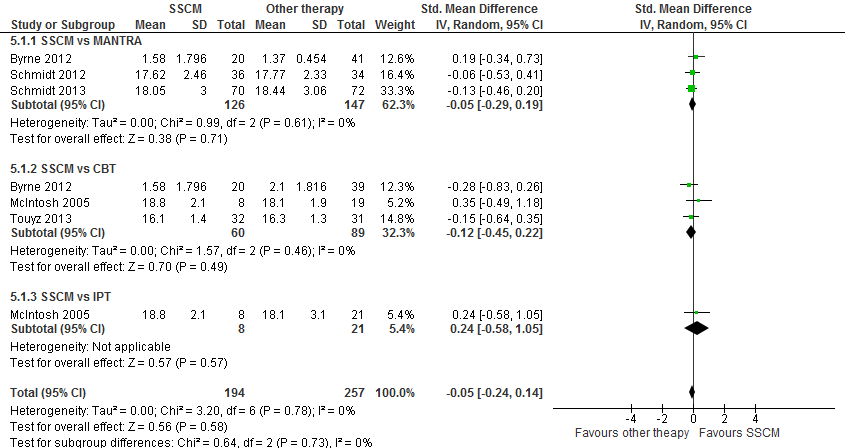


1. **SSCM versus another therapy, EDE global score or restraint (McIntosh et al., 2005)**


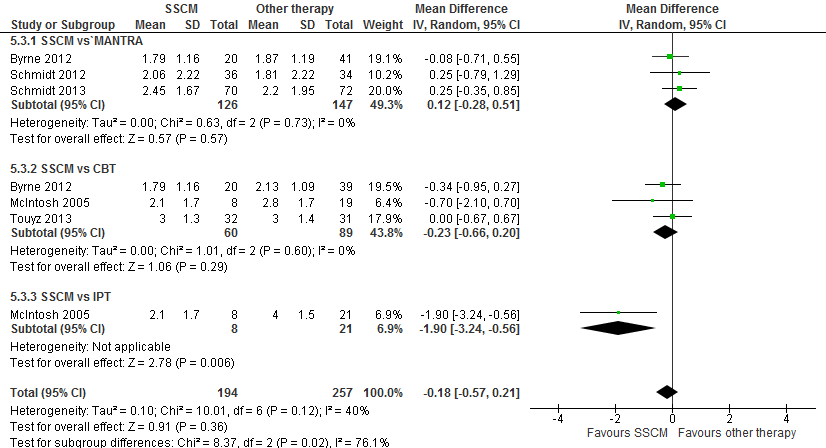


1. **SSCM versus another therapy, Attrition outcome**


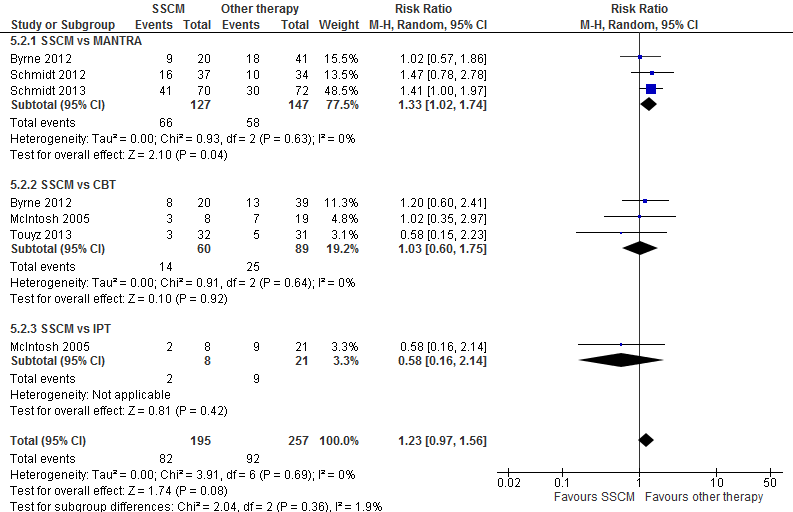

Supplement: Supplementary file 1 — Additional file 1. Secondary Analysis. [file 40337_2022_557_MOESM1_ESM.docx]
